# Evaluation of the Influence of the Tool Set Overhang on the Tool Wear and Surface Quality in the Process of Finish Turning of the Inconel 718 Alloy

**DOI:** 10.3390/ma17184465

**Published:** 2024-09-11

**Authors:** Krzysztof Smak, Piotr Szablewski, Stanisław Legutko, Jana Petru, Jiri Kratochwil, Sylwia Wencel

**Affiliations:** 1 Faculty of Mechanical Engineering, Poznan University of Technology, 3 Piotrowo Street, 60-965 Poznan, Poland; 2 Pratt & Whitney Kalisz, 4a Elektryczna Street, 62-800 Kalisz, Poland; piotr.szablewski@prattwhitney.com; 3 Institute of Mechanical Engineering, University of Kalisz, 4 Nowy Świat Street, 62-800 Kalisz, Poland; 4 Faculty of Mechanical Engineering, VSB—Technical University of Ostrava, Poruba, 708 00 Ostrava, Czech Republic; jana.petru@vsb.cz (J.P.); jiri.kratochvil@vsb.cz (J.K.); 5 Faculty of Production Engineering and Materials Technology, Czestochowa University of Technology, Av. Armii Krajowej 19, 42-201 Czestochowa, Poland; sylwia.wencel@pcz.pl

**Keywords:** Inconel 718, turning, surface roughness, tool wear, surface topography

## Abstract

The work deals with the influence of the reach of the applied tool holder on the edge wear, dimensional accuracy and surface quality defined by the topography as well as the roughness of the machined surface. The research has been conducted on specimens made of Inconel 718 in the configuration of sleeves, within the scope of finish turning with constant cutting parameters, *v_c_* = 85 m/min; *f* = 0.14 mm/rev; *a_p_* = 0.2 mm. The material under machining has undergone heat treatment procedures such as solution treatment and precipitation hardening, resulting in a hardness of 45 ± 2 HRC. Two kinds of turning holders have been used with the reaches of 120 mm and 700 mm. The tools are intended for turning external and internal surfaces, respectively. The tests have been conducted using V-shaped cutting inserts manufactured by different producers, made of fine-grained carbide with coatings applied by the PVD (Physical Vapour Deposition) and CVD (Chemical Vapour Deposition) methods. The edge wear has been evaluated. The value of the achieved diameter dimensions has also been assessed in relation to the set ones, as well as the recorded values of surface roughness and the surface topography parameters have also been assessed. It has been determined that the quality of the manufactured surface evaluated by the 2D and 3D roughness parameters, as well as the dimensional quality are influenced by the kind of the applied tool holder. The influence is also visible considering the edge wear. The smallest values of the deviations from the nominal dimensions have been obtained for the coated inserts of the range of higher abrasion resistance (taking into account information from the producers). The obtained results show that in predicting the dimensional accuracy in the process of turning Inconel 718 alloy with long-overhang tools, one should consider the necessity of correction of the tool path. Taking into account the achieved surface roughness, it should be pointed out that not only the kind of the tool coating but also the character of its wear has a great influence, particularly, when a long cutting distance is required.

## 1. Introduction

The aircraft industry certainly belongs to the most required ones, both with respect to the materials used and in respect to the technological solutions applied to obtain the required quality of components. One of the aircraft elements that require high execution quality is the engine because it has a great influence on flight safety. The occurrence of high temperatures in the aircraft engine determines the use of heat-resistant superalloys which maintain their properties under such conditions. Inconel 718 is one of these kinds of materials. It is widely applied in the aircraft industry, chemical industry, as well as in the nuclear industry [1]. It is part of a group of heat-resistant alloys on nickel matrix and is distinguished by high strength [2]. In addition to the aforementioned strength, the material has also remarkable hardiness to oxidation and corrosion, as well as creep resistance at high temperatures. The mentioned properties make it very commonly used in the production of components operating in the hot area of aircraft engines [3]. Such components include the main shafts of aircraft engines. This type of part, due to its great slenderness, is characterized by additional difficulties during the manufacturing process. These inconveniences are often related to the necessity of using tools with long overhangs during the shaping of inner surfaces. The distinguishing features of Inconel 718 are also low thermal conductivity, thermal diffusivity and its ability to strain hardening, which is why it is considered to be a difficult-to-cut material [4].

Aircraft engine components made of this alloy often face high technological demands. In most cases, they are related to obtaining the required dimensional accuracy and adequate surface quality [5]. Therefore, an assortment of adequate machining parameters and adequate tools is of crucial importance to ensure the stability of the machining process. Elaborating on the manufacturing strategy for components made of Inconel 718, one should keep in mind that in the process of cutting the material, the elevated temperature is generated in the area of the tool-workpiece interface. Such conditions, in combination with the mechanical loading of the edge, frequently cause rapid wear of the tool’s cutting edge [6].

Enterprises focused on the production of aircraft engine parts emphasize permanent improvement of their technological processes. Great attention is paid to the elaboration of a machining process making it possible to perform an operation for a given part without an operator’s participation. Such a philosophy of production organization determines the requirement of precisely defined tool life. This criterion is particularly significant in relation to the parts made of Inconel 718 because tool wear is much quicker here than in the case of machining other materials [7]. A prematurely worn edge influences the obtained dimensional, shape accuracy, the quality of the surface finishing and the features of the top layer. Bhatt et al. [8] have depicted the findings of experimental investigation of the configurations of the wear of functional coating of tungsten carbide (WC), tools with one-layer coating (TiAlN) PVD (Physical Vapour Deposition) and three-layer coating (TiCN/Al_2_O_3_/TiN) CVD (Chemical Vapour Deposition) when finish turning of Inconel 718. The authors have evaluated the rate of the edge wear and its mechanisms for the cutting speed (*v_c_*) range of 50–100 m/min and the range of feed (*f*) of 0.075–0.125 mm/rev with constant cutting depth (*a_p_*) of 0.25 mm. It has been found that abrasion and adhesion wear were the most predominant mechanisms of the wear-controlling destruction and ultimate collapse of the WC tool. It has been assessed that the three-layer (TiCN/Al_2_O_3_/TiN) CVD coating shows the highest wear hardiness at a high cutting speed of 100 m/min and at a low feed. Uncoated tools have ensured the best productivity at relatively low cutting speed levels (50 m/min) and feed in the range of medium value. A tool with one-layer PVD coating (TiAlN) has better wear resistance than other tools at an average cutting speed of 75 m/min. Khan et al. [9] have shown the results of tests after finishing turningthe Inconel 718 with the use of PCBN (Polycrystalline Cubic Boron Nitride) inserts with low content of boron nitride. The tests were performed with the use of orthogonal Taguchi matrix L36 which evaluated the influence of the cutting insert geometry (round, type C), preparation of the tool edge (grinding, chamfering, honing), fluid pressure (10, 100 bar), insert coating (uncoated, TiAlN + TiN), cutting speed (150, 300, 450 m/min) and the feed (0.05, 0.10, 0.20 mm/rev) with constant cutting depth of 0.2 mm on the tool life. It has been shown that, at the lowest cutting speed (150 m/min), the average tool life was about five times longer with the use of a round insert as compared to the type C tool. The flank wear was the dominant kind of wear in most tests as an outcome of abrasion; however, workpiece material build-up was similarly significant. When the cutting speed rose up to 300 m/min, the presence of grooves and BUE decreased, which led to comparable productivity between the C-type tools and the round ones. However, during increasing the speed up to 450 m/min, disastrous cracks of the insert took place, as well as chipping and thermal cracks, with tool life not exceeding 3.5 min. The graphs of the main effects have indicated that the use of round inserts without coating, with preparation of E25 cutting edge at the cutting speed of 150 m/min, feed of 0.05 mm/rev and fluid pressure of 10 bars has given the longest tool life. ANOVA calculations have shown that the cutting speed, feed and tool geometry had a meaningful influence on the durability of the tool with respective PCR 36.6%, 17.3% and 11.5%. Although it was not a statistically important factor, the use of cooling fluid under high pressure (100 bars) was sometimes harmful to tool life, especially at low cutting speeds. Fang et al. [10] have examined the effect of the tool cutting edge wear on the cutting forces and vibrations while finishing turning Inconel 718 at high speeds. The authors have conducted a series of experiments of turning with cutting inserts whose radii of the cutting edge rounding had various values in the scope of 2 to 62 µm. The research has proved that, in turning Inconel 718 at high speeds, the profile of the cutting insert edge changes dynamically in individual points of the tool cutting edge. Due to various cutting conditions and various initial values of the cutting edge profile at each point, the tool cutting edge wear in individual points follows different schedules of variability. The wear of the tool cutting edge in 3D machining is more composite than in the case of orthogonal 2D machining. Tests have shown that the edge wear grows with the increase in the cutting-edge rounding radius. In the case of the same cutting insert, the wear along the edge grows from the end point of the tool (apex-corner) towards the location close to the outer diameter of the workpiece under machining due to the bigger undeformed thickness of the chip and more plastic deformation of the material under machining which occurs in the shear zone. With the increase in the tool cutting edge wear during the process of machining, all three cutting force components grow. With the increase in the initial value of the cutting edge rounding radius, all three cutting force components grow. Compared to the other two, the growth of the feed component of the cutting force is the quickest. The traditional Fourier technique of quick transformation does not reveal the signals of vibration in a wide frequency range as the tool edge wear progresses. That is why this technique is not the best one for the analysis of machining vibrations that accompany tool tip wear dynamics.

As compared to Fourier transform, discrete wavelet transform is more effective in disclosing the changeability of machining vibrations in a wide frequency range. The discrete wavelet transform is a technique that is more suitable for the analysis of vibration in machining. This technique has also shown that the vibration amplitude rises with the increase in the cutting-edge rounding radius. Xavior et al. [11] have studied the effect of cutting conditions on the material under machining and the tool material during the machining of the Inconel 718 alloy. Those investigations were conducted for the purpose of better understanding the aspects of machinability, such as surface integrity, cutting forces, work hardening as a result of machining, as well as tool life and wear. The test results show that uncoated tools made of sintered carbides have worked properly in the cutting speed area of 10–30 m/min, at speeds of over 40 m/min, a better solution is coated tools of sintered carbides. This review article concerns also issues related to the assortment of the tool material, cutting environment and edge geometry. The tests have shown that the MQL cooling system renders minimum values of residual stresses for all the tested inserts at all the cutting speeds. The lowest surface roughness has been obtained for a sintered carbide insert at the cutting speed of 100 m/min and in the conditions of flood cooling. In the case of machining with high speeds, CBN and ceramic inserts reinforced with whiskers are more effective with respect to the tool life. The tests have also shown that the system of flood cooling causes a reduction in the cutting forces induced during machining and results in smaller microhardness of the elements undercutting. In terms of tool life, a cryogenic system of cooling is the best one. The surface roughness values are optimum at the cutting speeds of 30–40 m/min and in the feed range of 0.05–0.1 mm/rev. The microhardness value was 525 HV for dry machining and 510 HV for wet machining. With the increase in depth, hardness gradually declines and, at the depth of 250 µm reaches the volumetric value. Cantero et al. [12] have tested the productivity of PCBN tools from four different producers and a new kind of material, JP2 (bidemic) of NTK company (Tokyo, Japan) corresponding to the properties of CBN tools in finish turning of the Inconel 718 material under dry conditions. The authors have performed tests under various cutting conditions evaluating the evolution of the cutting forces, surface roughness and edge wear. In order to determine the reference conditions, a tool made of sintered carbides has also been tested. PCBN tools were tested in the range of parameters, *v_c_* = 200 m/min; *f* = 0.15 mm/rev; *a_p_* = 0.15 mm; *a_p_* = 0.25 mm; and *a_p_* = 0.5 mm. The sintered carbide tool has reached a life of up to 29 min at low cutting speeds in the range of 35–50 m/min and, therefore, the authors have confirmed the purposefulness of dry finish machining of Inconel 718 by this tool. For all the tests performed with tools of PCBN and JP2, the most frequent kind of wear was adhesion, chipping, notch and crater. From all the kinds of wear notch was the most frequent reason for the tool replacement. Under all the cutting conditions, the tool life was shorter than 2 min. The authors have stated that dry machining of Inconel 718 at high cutting speeds is not profitable in the industry. It should also be pointed out that enhancement of the cutting depth also leads to a reduction in the tool life. One should also keep in mind that, in the case of PCBN inserts, when the wear is located on the tool vertex or along the cutting edge, the roughness is above 2 µm. Devillez et al. [13] have proved that a tool coated with AlTiN, on account of its high hardness (3300 HV 0.05), compared to other coated tools, had proper anti-friction features and less abrasion wear. The authors have indicated, however, that on account of a short time of machining, only the initial stage of wear has been investigated. In this phase, flank wear has been observed, as well as notch wear. Costes et al. [14] have investigated the influence of the cutting speed on the signs of the wear of cutting inserts made of CBN. They have proved that, at the higher values of cutting speed, i.e., above 250 m/min, the predominant wear types are adhesion, diffusion and finally abrasion. The authors have also shown that, with respect to wear, the best results in the tests were inserts with a content of 45% to 60% CBN and TiN ceramic binder and working at the cutting speed area of 250–300 m/min. D’Addona et al. [15] have studied the influence of elevated cutting speeds on the wear of the tool and surface roughness during cutting of Inconel 718 with sintered carbide inserts with CVD coating. The area of the investigated speeds was up to 266 m/min. When machining at the highest values of cutting speed applied in the investigation, the authors noticed significantly higher temperatures in the cutting space. The growth of the temperature has resulted in more wear of the cutting edge, the wear was 3.5 times bigger than that in the case of the speed of 90 m/min and 105 m/min. At elevated cutting speeds of 255 m/min and 190 m/min, besides very significant flank wear, burns have also been found. On the other hand, at the speeds of 90 m/min and 105 m/min, the recorded wear is even along the corner radius of the insert, peeling of the coating has also been noticed. It should be noted that in that work the length of the cutting path was 28 m, whereas the authors of the present work have performed an investigation with a cutting path distance of 1600 m.

The previously presented works clearly prove, that the low heat conductivity of Inconel 718 results in a significant portion of the heat generated in machining penetrates the cutting tool [16]. According to the investigators, this is the major reason for the tool wear; however, the surface hardened from the anterior machining is responsible for the occurrence of notch wear.

Therefore, tool life is of decisive importance, particularly when adequate surface quality and dimensional accuracy are required. Companies specializing in the production of cutting tools for machining heat-resistant alloys adopt the edge life at the level of 15 min. The life depends on the rigidity of the MFWT (Machine, Fixture, Workpiece, Tool) system. Considering machining of thin-walled components vibrations may often occur. Its presence most frequently results in the deterioration of the tool’s life. Machining of parts with a big slenderness ratio, particularly ones that require the use of tools of long reach, may also be problematic. Reduced stiffness of the tool, as well as the occurring vibration, also contribute to shortening the tool’s life. They often result in difficulties in achieving the required dimensional accuracy and surface quality.

In the case of finish machining, investigations concerning the life of edges recommended for machining nickel-based alloys are most often focused on coated tools of sintered carbides [17]. Tool coatings are generally applied using the PVD or CVD method (Physical Vapour Deposition or Chemical Vapour Deposition, respectively).

The application of proper parameters of machining, i.e., cutting speed, feed and depth of cut, as well as a selection of a tool with adequate geometry and coating have a great influence on the edge wear. This, as a consequence, influences the quality of the produced area and the accuracy of geometric features which can be evaluated by measurement of roughness and examination of the surface layer microstructure, but also by verification of the achieved dimensions [18,19].

The conducted analysis of research works indicates a research gap concerning the influence of a large tool overhang on the wear of the cutting edge, and consequently, on the topography of the obtained surface. In all analyzed studies, researchers used external turning tool holders, which are short and rigid. The use of a long tool holder reduces this rigidity. This rigidity affects not only dimensional accuracy but also cutting-edge wear and topography. Additionally, the studies employed cutting inserts with coatings applied by PVD and CVD methods, which are characterized by different chemical compositions and hardness, translating into resistance to abrasive wear. The purpose of this research was to evaluate the wear of cutting edges and the topography of surfaces obtained after longitudinal turning using tool holders with small and large overhangs. Additionally, this research also aims to contribute to the improvement of the finish turning process for the internal surface of a thin-walled aircraft engine shaft class part using a tool with a long overhang, where continuous tool operation time exceeding 15 min is required. The research results have significant practical importance as they were conducted in a facility that specializes in the production of main shafts for aircraft engines. These parts undergo significant loads during operation, hence they are subject to very high-quality requirements.

## 2. Materials and Methods

**Figure 1:**
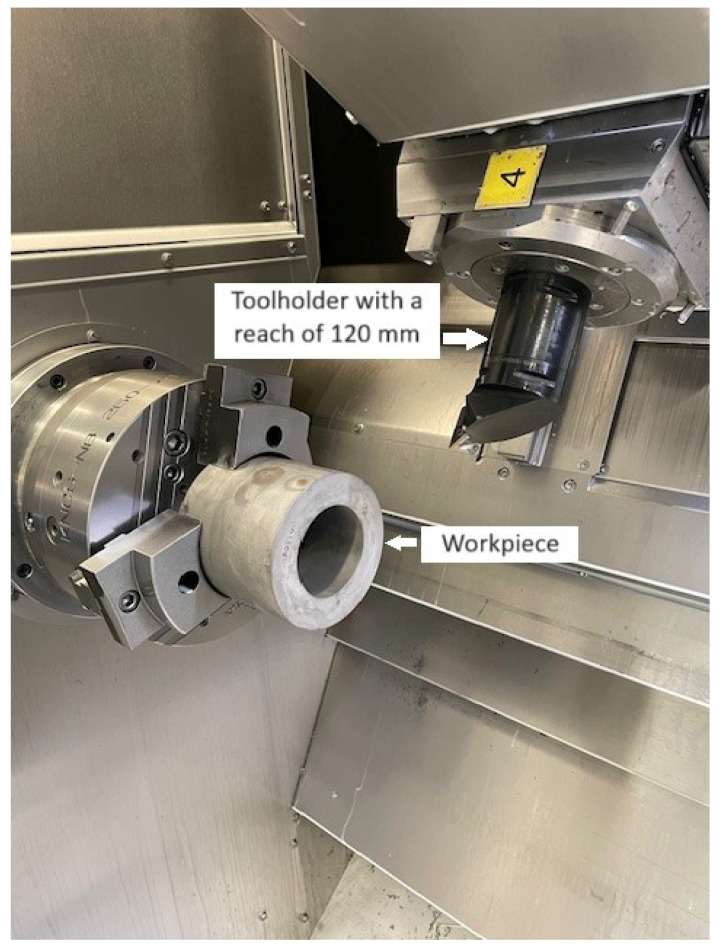
Holder C6-SVHBL-45065-16HPA and the specimen utilized in the tests.

**Figure 2:**
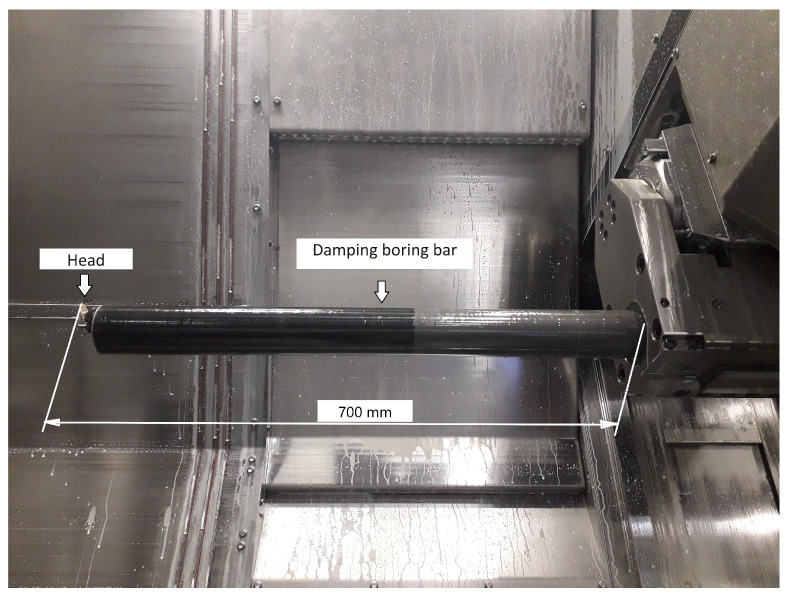
Anti-vibration holder S-570-3C-70/80-1053-40CR with the head SL-SVLBL-40-16HP.

Comparative studies were conducted on the WFL M40 multitasking turning-milling center. The material utilized in the tests was aged Inconel 718 with a hardness of 45 ± 2 HRC in the configuration of sleeves. The specimens had the following dimensions: external diameter of 132 mm, inner diameter of 77.4 mm and length equal to 122 mm. Five cutting inserts with positive geometry were used with a corner radius equal to 0.8 mm, supplied by various manufacturers. The tested tools were made of sintered carbides (Table 1). According to the information available in the producers’ catalogs, the above tools have high abrasion resistance. The research was conducted under conditions enabling the use of high pressure of the cooling and lubricating fluid, Ecocool Global 10, to be applied. The liquid is produced by Fuchs Oil Corporation (Gliwice, Poland). This is an 8% emulsion concentrate based on mineral oil and 92% of water. Standard Capto holder type C6-SVHBL-45065-16HPA (Figure 1) with a reach of 120 mm was used, as well as an anti-vibration boring bar S-570-3C-70/80-1053-40CR connected to a head, SL-SVLBL-40-16HP (Figure 2) with the reach of 700 mm. Those tools are destined for machining outer surfaces and holes, respectively. For the tool with a long overhang, in order to compensate for the deflection occurring on it, each time, after the insert was installed, the tool was automatically measured on the probe for tool measurement which is provided to the machine. Moreover, after the first pass, the conformance of the obtained dimension to the programmed dimension was checked, in case of discrepancy, the tool geometry was corrected.

**Figure 3:**
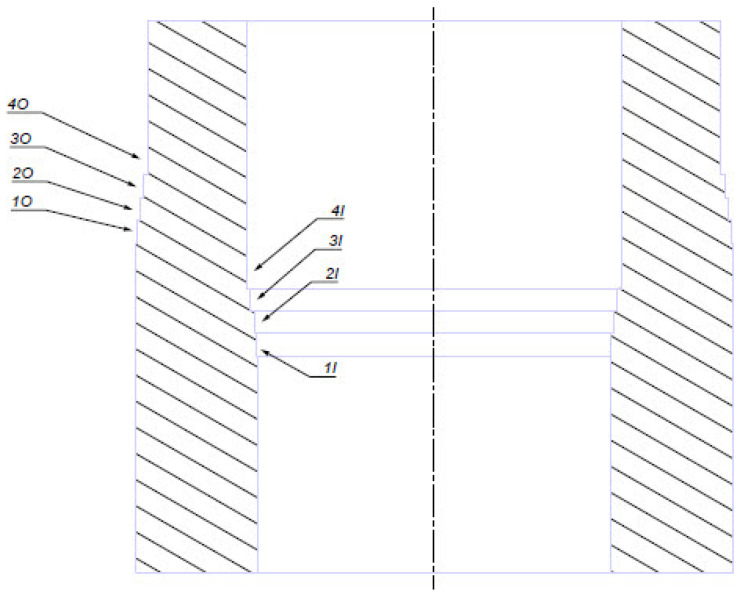
A view of a sample after the performed tests (1O—first surface outside, 2O—second surface outside, 3O—third surface outside, 4O—fourth surface outside, 1I—first surface inside, 2I—second surface inside, 3I—third surface inside, 4I—fourth surface inside).

The machining parameters have been selected on the basis of previous investigations: *v_c_* = 85 m/min; *f* = 0.14 mm/rev; and *a_p_* = 0.2 mm [3]. The pressure of the lubricating and cooling fluid was 80 bar. The research has been conducted with the application of longitudinal turning of the external and inner diameter. The present paper concerns an investigation aiming at elaboration of a technological solution making it possible to optimize the turning of inner surface parts type shaft, which requires the use of a tool with a reach of (10 x D). That is why each insert has performed 14 passes in both holders. The passes had lengths of 74–35 mm, depending on the holder. The total length of the passes reflected the needed spiral cutting path of one tool pass, which amounted to 1600 m. For each edge working in an individual holder four measurements of the achieved diameters after not randomly defined passes have been performed, reflecting inspection points for the machined surface of the production part, whose cutting path of the edge has been replicated. The drawing requirements for the machined area were the average surface roughness *Ra* = 1.6 µm and the tolerance of the hole diameter ± 0.05 mm. The strategy of the passes has been designed in this way that upon gaining the cutting path for the given checkpoint, the next turnings could be performed on a shortened segment in order to perform measurements of diameters, roughness and topography of the surface (Figure 3).

**Figure 4:**
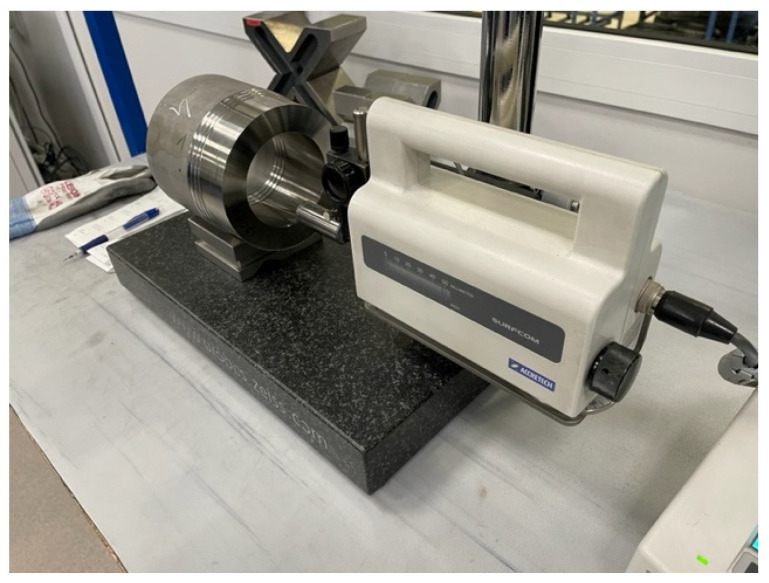
Measurement of the surface roughness.

The surface roughness has been evaluated using the *Ra* parameter which was measured at the same checkpoints as the diameter. For every specimen in a defined space, measurement was performed at three positions on the diameter located at intervals of 120 degrees. For this purpose, a Surfcom 130A profilometer from Seimitsu Company (Tokyo Seimitsu Co., Ltd., Tokyo, Japan) was used (Figure 4). The surface roughness parameters have been measured with l_r_ = 0.25 mm and l_n_ = 2 mm.

According to the above configuration, the topography of the machined surface has also been measured. Measurements were conducted with the Nanoscan 855 device from Jenoptik Company (Hommel–Etamic, Villingen-Schwenningen, Germany) and defined by the *Sa* parameter. The parameters of topography were measured with the use of a Gaussian filter on an elementary area with dimensions of 1.2 mm and 1 mm following ISO 25178 standards [20].

**Figure 5:**
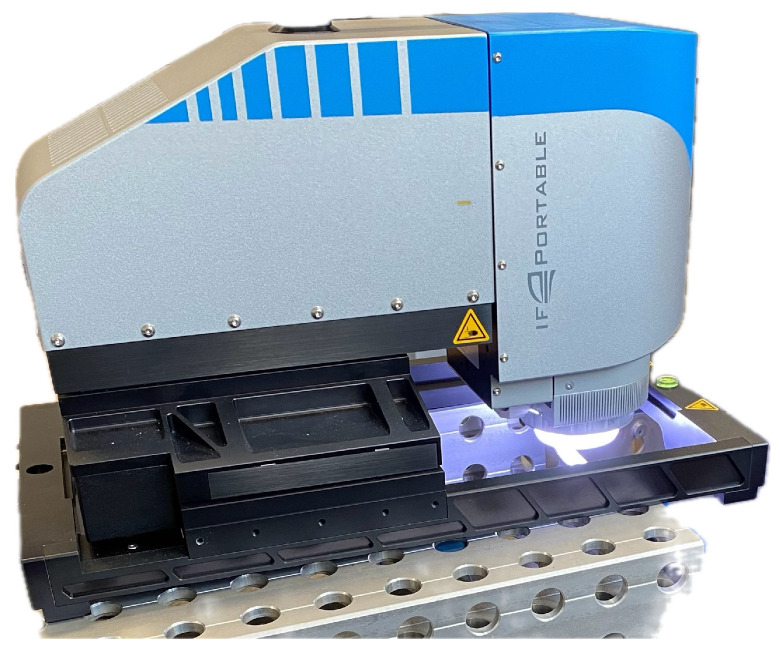
3D Portable RL optical microscope of Bruker Alicona Company.

The measurement of the edge microgeometry with special attention paid to the value of the cutting edge rounding radius (*r_n_*) of the new edges as well as the wear of the working edges was performed using a 3D Portable RL optical microscope made by Bruker Alicona Company (Graz, Austria) according to ISO 3685 standard (Figure 5) [21]. The measurements were conducted at a 10 × objective magnification, where the transverse measurement area was 4 mm^2^, the best transverse topographic resolution was 2 µm and the vertical resolution was 150 nm.

The obtained dimensions of the outer diameters were measured in two cross sections using an electronic micrometer No.: 293–251 with a measurement area of 125–150 mm of the Mitutoyo Company (Kawasaki, Japan). The dimensions of the inner diameters were measured using a three-point bar gauge No.: 5109207 with a measurement range of 70–85 mm. For the adopted cutting parameters, using Equations (1) and (2) from work [3], the theoretical–predictable value of the roughness parameter *Ra* was determined and is equal to *Ra* = 0.78 µm. Because, according to work [18], the two-dimensional parameter *R* determining surface roughness is considered equivalent to the *S* parameter in three-dimensional analysis, therefore, an identical predictive value was adopted for the *Sa* parameter. For the obtained measurement results of surface roughness and topography, scatter intervals have been calculated.

## 3. Results and Discussion

**Figure 6:**
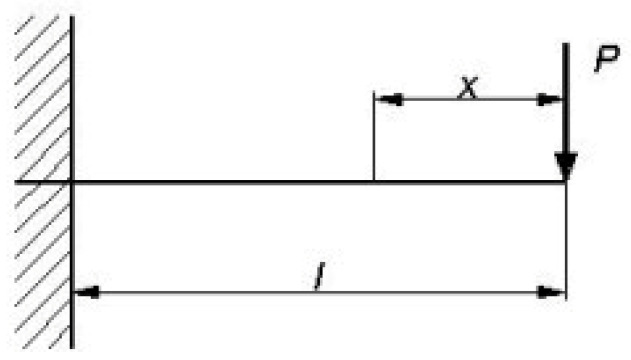
Auxiliary drawing for model calculations.

Since the authors have used a tool with long reach whose stiffness was certainly not at the same level as in the case of a standard Capto holder, its deflection during the tests had to be taken into consideration. It has been decided to model the value of the deflection taking advantage of the differential equation of the deflection line (1) for the case of a beam fixed on one side (Figure 6) [22]. The value of the passive force (this kind of force will have the greatest influence on deflection) has been assumed based on works [23,24] and [25], at the level of 200 N.

(1) $$EJ\frac{d^{2}y}{{dx}^{2}}=-Mg(x)$$

$$EJ\frac{d^{2}y}{{dx}^{2}}=-Px$$

where:

*D* = 70 mm = 0.07 m—*the tool diameter*
*R* = 35 mm = 0.035 m—*the tool radius*
*E* = 500 GPa = 500,000,000 kPa—*Young’s modulus for sintered carbides (the tool material)*
*J*—*moment of inertia*
*l* = 700 mm = 0.7 m—*not supported length of the tool*
*Mg*—*bending moment*
*P* = 200 N = 0.2 kN—*force*

First integration

$$EJ\frac{dy}{dx}=-P\frac{x^{2}}{2}+c=-\frac{1}{2}Px^{2}+c$$

Second integration

$$EJy=-\frac{1}{2}P\frac{1}{3}x^{3}+cx+d=-\frac{1}{6}Px^{3}+cx+d$$

Boundary conditions

1. If the tangent to the beam deflection line is horizontal at the fixed support point, then the derivative is equal to zero.

$$x=l\;y^{\prime }=0$$

2. The deflection at the fixed support point is equal to zero.

x=l y=0

1.

$$EJ0=-\frac{1}{2}Pl^{2}+c\;0=-\frac{1}{2}Pl^{2}+c\;c=\frac{1}{2}Pl^{2}$$

2.

$$EJ0=-\frac{1}{6}Pl^{3}+\frac{1}{2}Pl^{2}l+d\;0=-\frac{1}{6}Pl^{3}+\frac{1}{2}Pl^{3}+d\;0=-\frac{1}{6}Pl^{3}+\frac{3}{6}Pl^{3}+d\;0=\frac{2}{6}Pl^{3}+d\;d=-\frac{2}{6}Pl^{3}\;d=-\frac{1}{3}Pl^{3}$$

Equation of the deflection line

(2) $$EJy=-\frac{1}{6}Px^{3}+cx+d$$

$$y=-\frac{Px^{3}}{6EJ}+\frac{Pl^{2}x}{2EJ}-\frac{Pl^{3}}{3EJ}$$

Deflection

(3) $$y(x=0)=-\frac{Pl^{3}}{3EJ}$$

Calculation of the moment of inertia

$$J=\frac{\pi R^{4}}{4}$$

$$J=\frac{\pi 0.035^{4}}{4}$$

$$J=0.00000118\;m^{4}$$

Calculation of deflection

$$y\left(x=0\right)=-\frac{0.2\times 0.7^{3}}{3\times 500000000\times 0.00000118}=-\frac{0.0686}{1770}$$

$$y\left(x=0\right)=-0.000038\;m=-0.038\;mm$$

The results of deflection examined on the machine by the measurement of the first pass were at the level of 0.025–0.035 mm. It should be kept in mind that, after installing a new edge, the tool was measured each time. It must be pointed out, however, that the geometry of the inserts and the microgeometry of their edges differed from each other, which has influenced the discrepancy between the passive force value and the one assumed in the calculations. That is why the differences of the deflections tested on the machine in relation to the model value calculated using Equations (2) and (3) differ by up to 34%.

**Figure 7:**
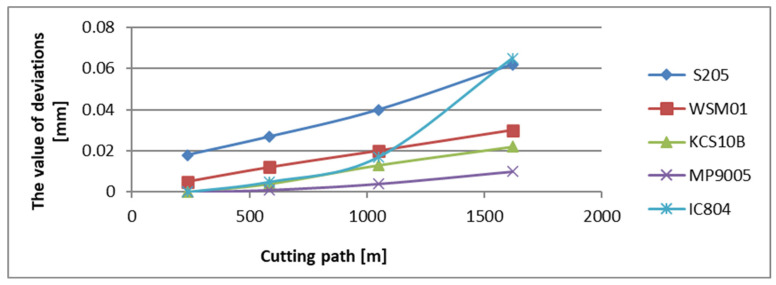
The deviation values from the nominal dimension in relation to the cutting path for the edges examined in a tool holder with a reach of 120 mm.

The dimensional accuracy and surface quality were evaluated based on the applied tool holder and the character of the edge wear. The surface quality was evaluated by using the surface roughness parameter *Ra*, and the stereometry parameter *Sa.* The accuracy of the achieved dimensions was controlled by measurement of the outer diameters using an electronic micrometer, the inner diameters were checked with a three-point gauge. Each edge on the individual holders has executed 14 passes and, consequently, the spiral cutting path of 1620 m has been received [26]. For the assumed strategy and cutting parameters, the operating time of each edge was 19 min. The diameter measurement was not randomly realized after the 2nd, 5th, 9th and 14th pass, respectively for each tool (Table 2 and Table 3). Analyzing the influence of the applied tool holder reach on the obtained dimensional accuracy in longitudinal turning, it can be observed that the length of the toolset plays a great role in keeping the required dimension during the tool operation. The results below show that, after passing the required cutting path, the smallest value of deviation from the nominal dimension for the holder with the reach of 120 mm has been noted for an area machined with an MP9005 edge and it was 0.01 mm. The greatest values of dimensional deviations have been observed for surfaces achieved as a result of machining with S205 and IC804 edges and they were 0.062 mm and 0.065 mm, respectively (Figure 7). For the holder with the overhang of 700 mm, the deviation from the nominal dimension grew and its smallest value was 0.21 mm for a surface machined with an S205 edge.

**Figure 8:**
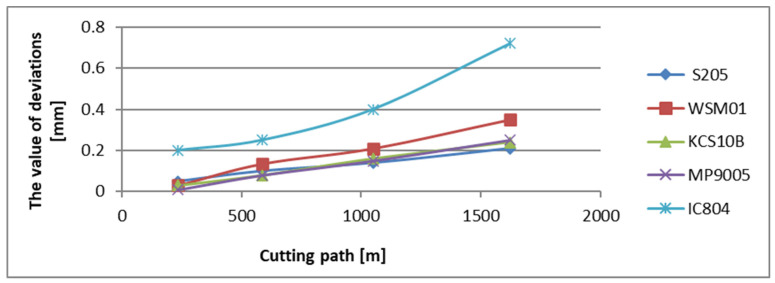
The deviation values from the nominal dimension in relation to the cutting path for the edges examined in a tool holder with a reach of 700 mm.

The greatest deviation from the dimension has been recorded for the surface cut with insert IC804, which amounted to 0.72 mm. The deviation values from the nominal dimension for the surfaces machined with KCS10B and MP9005 inserts show a similar level and amount of 0.24 mm and 0.25 mm, respectively (Figure 8).

**Figure 9:**
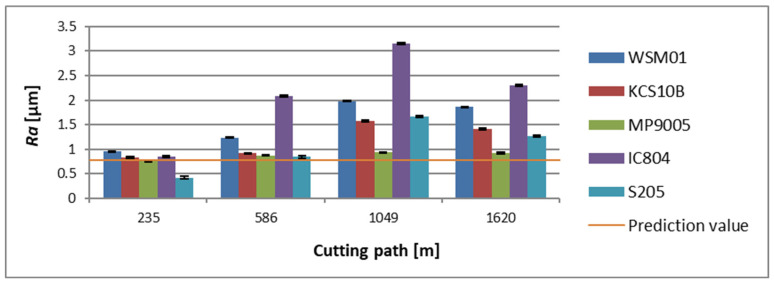
The obtained value of the surface roughness parameter *Ra*, in relation to the cutting path for the edges examined in a holder with a reach of 120 mm.

Considering the influence of the holder reaches on the surface roughness evaluated by the *Ra* parameter, it should be noted that it was checked in points referring to the tested dimensional accuracy. In addition to the holder stiffness, one should also consider the cutting-edge rounding radius whose value, according to Nieslony et al. [27], influences the roughness of the machined surface because it influences the minimum thickness of the chip. In the case of both holders, it can be seen that the value of roughness has developed with the progress of the cutting path traversed by the edge. For the holder with the overhang of 120 mm, the lowest average value of the *Ra* parameter at the initial phase of machining was registered for a surface processed with the S205 insert and it was *Ra* = 0.423 µm. However, the highest average value of roughness at that stage was recorded for a surface obtained by machining with a WSM01 insert, which amounted to *Ra* = 0.957 µm. Considering the final phase of machining for the tool under discussion, the lowest mean value of the *Ra* parameter has been recorded for a surface cut with the MP9005 insert and it was *Ra* = 0.924 µm. The surface showing the highest average roughness value was one achieved as a result of machining with an IC804 insert, its roughness was *Ra* = 2.302 µm (Figure 9).

**Figure 10:**
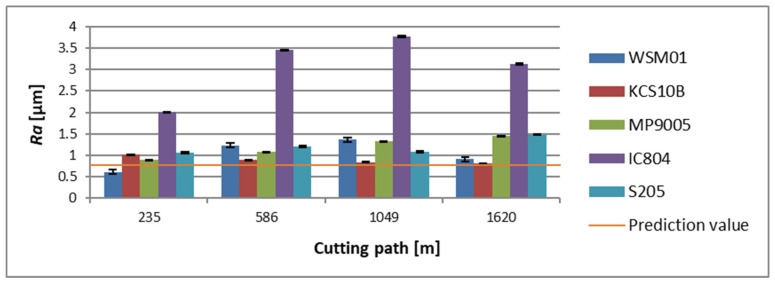
The obtained value of the surface roughness parameter *Ra*, in relation to the cutting path for the edges examined in a holder with a reach of 700 mm.

For the holder with a 700 mm overhang, at the initial phase of machining, the lowest average *Ra* parameter value was registered for an area machined with a WSM01 insert, where *Ra* = 0.610 µm. The highest average roughness value at that stage was noted for an area machined with an IC804 insert, for which *Ra* = 2.003 µm. Taking into consideration the final phase of machining for the present tool, the lowest mean value of the *Ra* parameter was achieved for a surface machined with a KCS10B insert, which amounted to *Ra* = 0.804 µm. However, the highest average roughness value was noticed for an area machined with an IC804 insert, where *Ra* = 3.133 µm (Figure 10).

**Figure 11:**
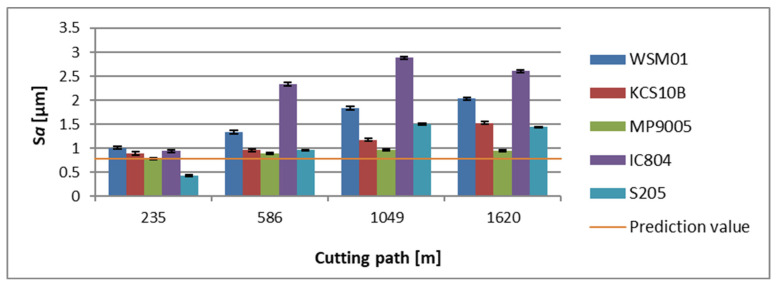
The obtained value of the surface topography parameter *Sa,* in relation to the cutting path for edges examined in a holder with a reach of 120 mm.

Analyzing the impact of the tool reach on the surface topography evaluated by the *Sa* parameter, one can observe that the measurement results are at a similar level as in the case of roughness measurements [28,29]. For the holder with the overhang of 120 mm, the lowest average value of the *Sa* parameter at the initial stage of machining has been recorded for a surface that had been machined with an S205 insert and it was *Sa* = 0.436 µm. The highest average topography value at that stage was achieved for a surface resulting from machining with a WSM01 insert and it was *Sa* = 1.01 µm. As regards the terminal phase of machining for this tool, the lowest average value of the *Sa* parameter was recorded for an area cut with an MP9005 insert and it was *Sa* = 0.952 µm. The surface characterized by the highest average value of this parameter was one cut with an IC804 insert, having *Sa* = 2.606 µm (Figure 11).

**Figure 12:**
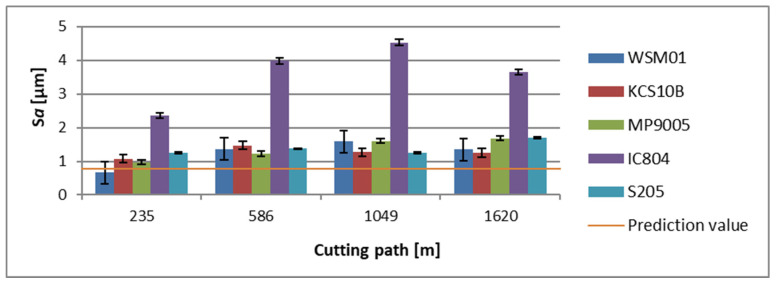
The obtained value of the surface topography parameter *Sa,* in relation to the cutting path for edges examined in a holder with a reach of 700 mm.

For a tool holder with a reach of 700 mm, at the initial phase of machining, the lowest average *Sa* parameter value was registered for an area machined with a WSM01 insert, where *Sa* = 0.662 µm. However, the highest average topography value at this stage of machining was noticed for an area machined with an IC804 insert, for which *Sa* = 2.36 µm. As regards the final phase of machining for the tool under discussion, the lowest average *Sa* parameter value was recorded for a surface cut with a KCS10B insert and it was *Sa* = 1.253 µm. However, the area characterized by the highest average value of this parameter was one cut with an IC804 insert, whose *Sa* = 3.656 µm (Figure 12).

It should be kept in mind that in all the cases, the *Sa* parameters have higher values as compared to the *Ra* parameters which is a result of the size of the tested area. The *Sa* parameter was examined in an area, not linear way and consequently, it comprises a bigger spectrum of surface irregularities. Taking both holders into account, the highest values of the *Ra* and *Sa* parameters at the final phase of machining were recorded for surfaces machined with the edges of the IC804 insert. It has to be pointed out, that those edges had the highest values of the cutting-edge rounding radius, which was partly the reason for achieving the highest value of the above parameters. It should also be stated that, in the progress of machining time, in contrast to the achieved dimensional accuracy, in the case of surface roughness, lower values of it are recorded for some edges (WSM01, KCS10B) working in a holder with the overhang of 700 mm. This is due to the fact that a shorter holder ensures greater rigidity of the MFWT system resulting in larger loads being transmitted directly to the cutting edge. On the other hand, the tool holder with a longer overhang but provided with a vibration damping system takes over a part of the loads by its previously mentioned deflection. This results in smaller flank wear in the case of some edges and it alters the character of the wear of the edge, which influences the resulting surface roughness.

**Figure 13:**
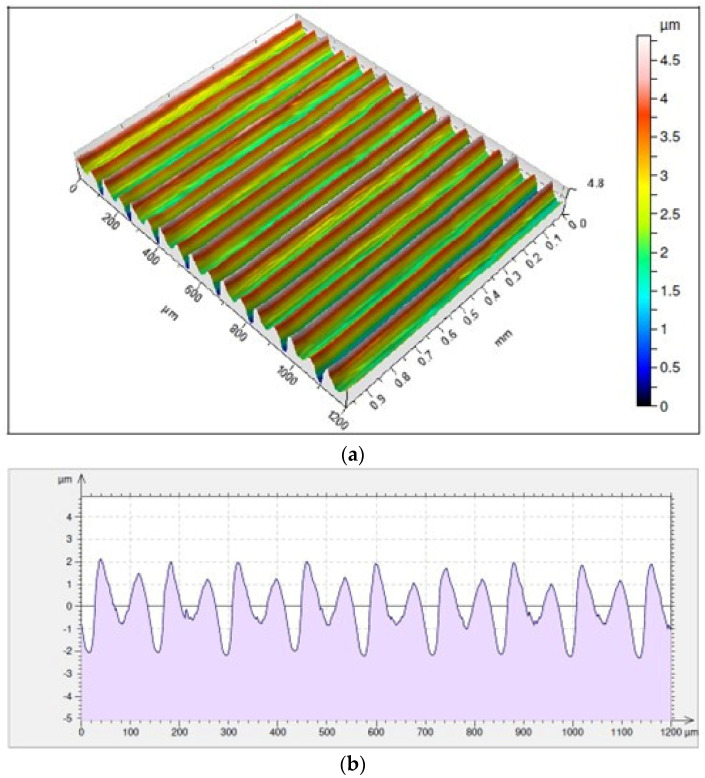
Surface topography and roughness achieved with a VBMT 160408-LS MP9005 insert in a tool holder with an overhang of 120 mm: (**a**) 3D surface topography, (**b**) 2D surface roughness profile.

Analysis of isometric maps of the investigated surfaces shows an even distribution of valleys and peaks in the case of machining with a tool holder with an overhang of 120 mm. In the case of the holder with 700 mm reach, the distribution is not as uniform, which is on account of the less stability of the cutting process. In most cases, those surfaces have various heights of micro-irregularities. The isometric maps discussed in the further part concern surfaces from the final phase of machining when the covered cutting path of the edge comes close to the value of 1600 m. In order to detail the analyzed maps, their two-dimensional charts were also presented. For the holder with the overhang of 120 mm, the lowest value of the height of irregularities was registered for a surface machined with an MP9005 insert for which *Rz* = 4.03 µm. For this edge, parameter *Rp*, i.e., the maximum height of the surface profile peaks had slightly lower values as compared to the *Rv* parameter, defining the maximum height of the surface profile valleys. The average values of those parameters were *Rp* = 1.90 µm and *Rv* = 2.12 µm (Figure 13).

**Figure 14:**
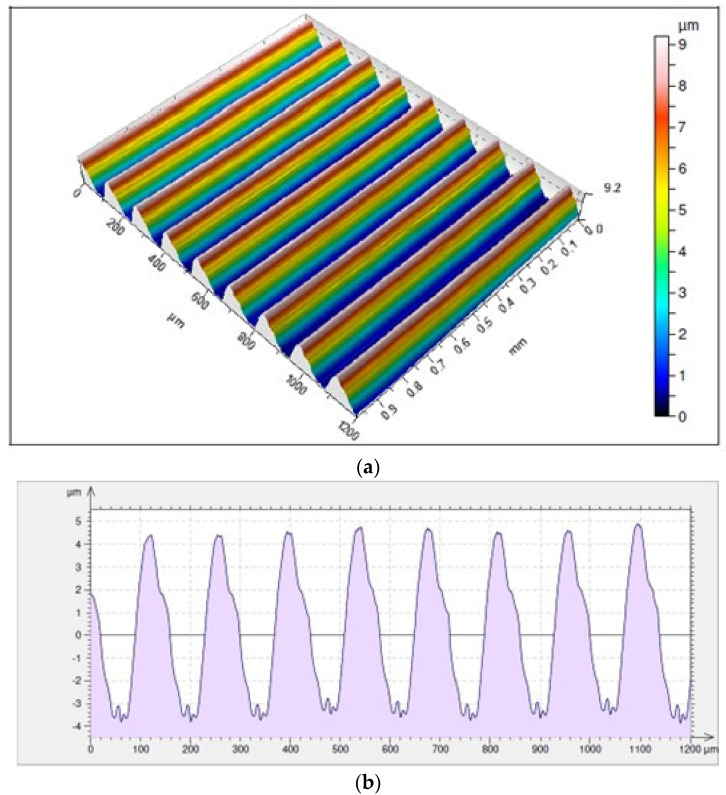
Surface topography and roughness achieved with a VCMT 160408–SM IC804 insert in a tool holder with an overhang of 120 mm: (**a**) 3D surface topography, (**b**) 2D surface roughness profile.

**Figure 15:**
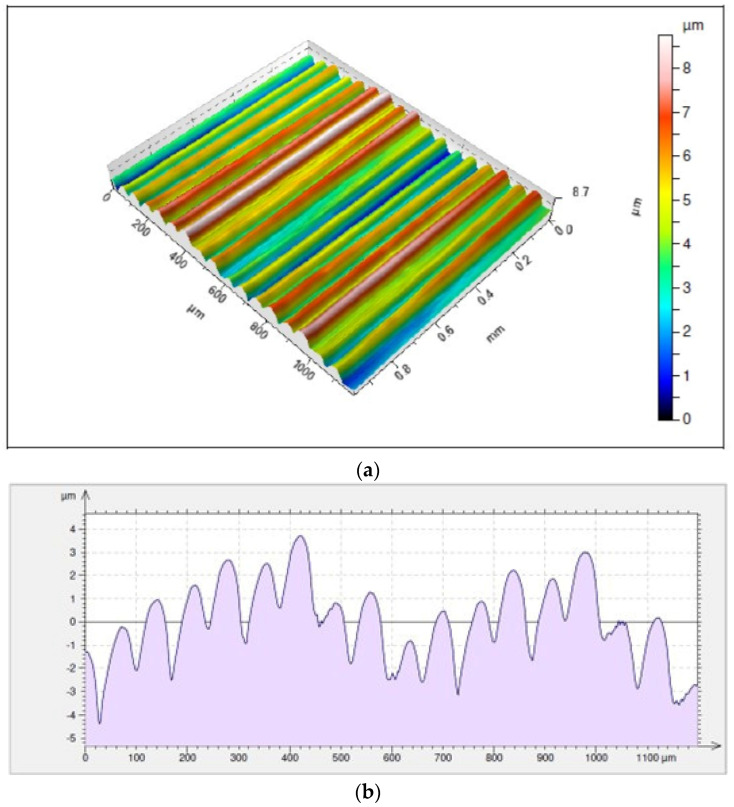
Surface topography and roughness achieved with a VBGT 160408LF KCS10B insert in a tool holder with an overhang of 700 mm: (**a**) 3D surface topography, (**b**) 2D surface roughness profile.

The surface for which the highest average value of the height of irregularities has been recorded is the one achieved as a result of cutting with an IC804 insert, where *Rz* = 7.41 µm. This surface shows a much higher value than the parameter *Rp* = 4.13 µm as compared to *Rv* = 3.28 µm (Figure 14). Taking into consideration the holder with a reach of 700 mm, the lowest average value of the height of irregularities was noticed for an area machined with a KCS10B insert, where *Rz* = 3.77 µm. This surface, compared to that cut with a holder of a shorter reach for which the lowest values of the *Rz* parameter were noticed, is characterized by much lower average values of the *Rp* parameter as compared to *Rv* whose results are as follows: *Rp* = 1.55 µm and *Rv* = 2.22 µm (Figure 15).

**Figure 16:**
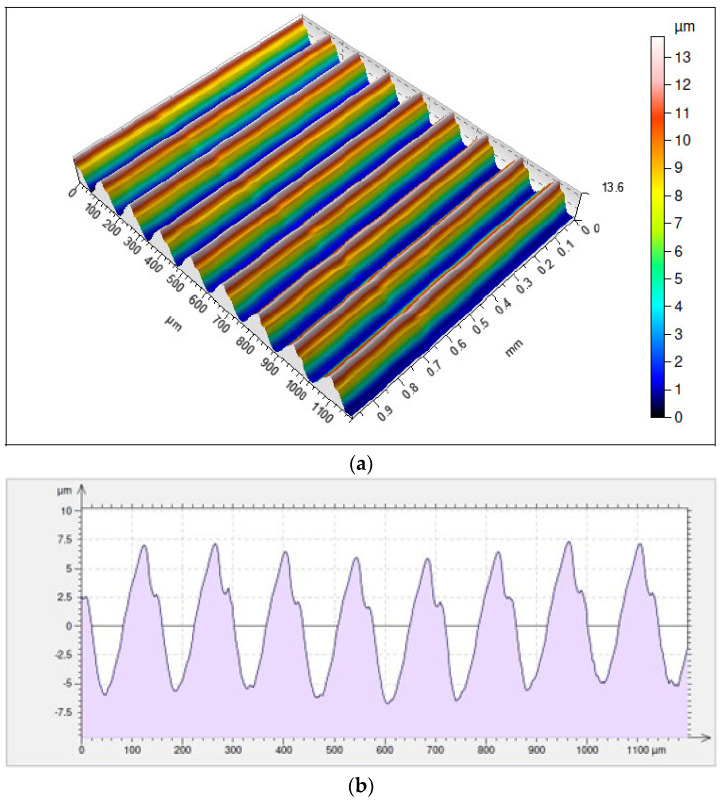
Surface topography and roughness achieved with a VCMT 160408–SM IC804 insert in a tool holder with an overhang of 700 mm: (**a**) 3D surface topography, (**b**) 2D surface roughness profile.

The highest value of the height of irregularities of the surface obtained with the use of the holder under discussion was noted for the surface resulting from cutting with an IC804 insert where *Rz* = 11.4 µm. The differences in the average values of the parameters *Rp* and *Rv* are not significant in this case and they are *Rp* = 6.03 µm and *Rv* = 5.32 µm (Figure 16).

In the case of both holders, it can be noticed that the surfaces cut with the IC804 edges that, at the final phase of machining, the *Rp* parameter values were higher as compared to the *Rv* parameters. Considering tribological and functional aspects, this is a disadvantageous feature of the topography or roughness profile, promoting the increase in the factor of void *Sp*/*Sz* and equivalently *Rp/Rz*, as mentioned by Pawlus et al. [30].

**Figure 17:**
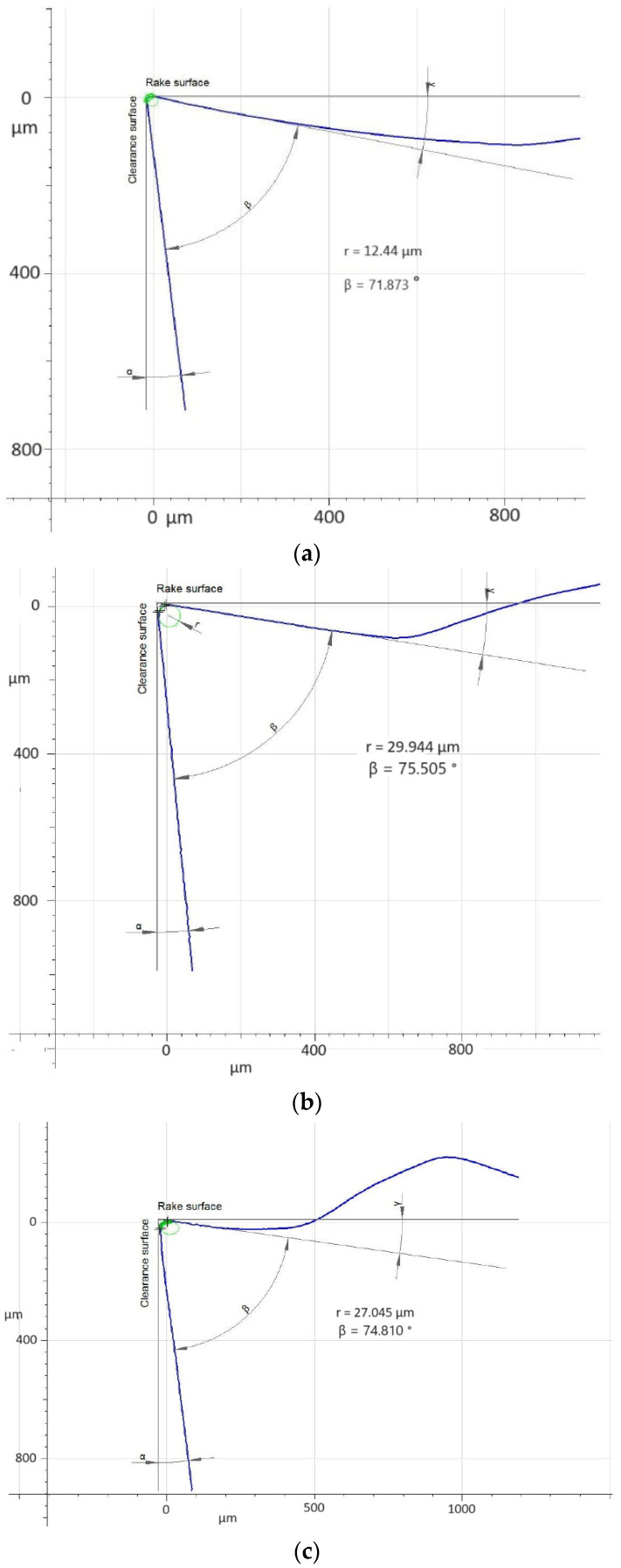

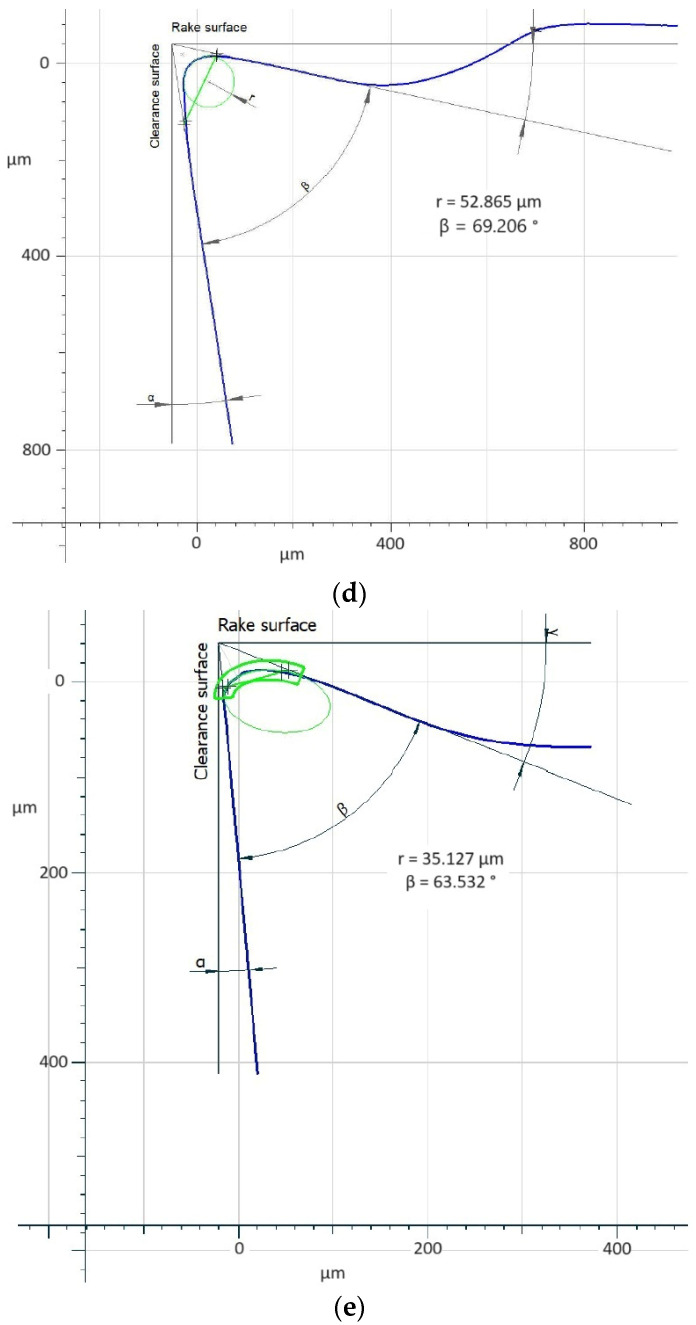
Comparison of the micro-geometry of new cutting edges with special consideration of the radius of the cutting edge rounding *r_n_*: (**a**) the edge of insert VCGT160408–MM4 WSM01 (Walter) *r_n_* = 12.44 µm, (**b**) the edge of insert VBGT 160408LF KCS10B (Kennametal) *r_n_* = 29.944 µm, (**c**) the edge of insert VBMT 160408–LS MP9005 (Mitsubishi) *r_n_* = 27.045 µm, (**d**) the edge of insert VCMT 160408–SM IC804 (Iscar) *r_n_* = 52.865 µm, (**e**) the edge of insert VBGT 160408–UM S205 (Sandvik) *r_n_* = 35.127 µm.

Evaluating the influence of the reach of the applied tool holder on the magnitude and character of the edge wear, it should be pointed out that each of the tested tools was working for about 19 min. In the case of Inconel 718 material, such working time exceeds the basic recommendations for the edge life equal to 15 min, as suggested by the manufacturers of cutting tools. It should be stated that the durability of the tool, in addition to its stiffness, is influenced by the kind of edge coating and the method of its application, as well as its micro-geometry (Figure 17). Considering the width of the flank wear evaluated with the *VB_C_* parameter and the view of the edge itself, one can see a significant difference between the tools working in holders of different stiffness.

**Figure 18:**
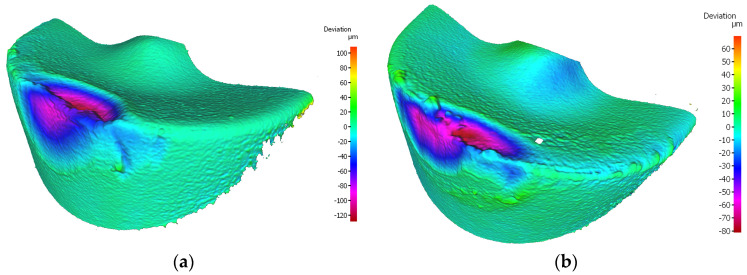
Topography of the worn cutting edge of the insert VCMT 160408–SM IC804 after 19 min. of machining: (**a**) an edge from a holder with a reach of 120 mm, (**b**) an edge from a holder with a reach of 700 mm.

The highest value of the width of the flank wear of the edges working in a holder with a reach of 120 mm was recorded for the insert IC804 for which *VBc* = 0.452 mm. Considering the tool holder with a 700 mm overhang, this edge had also the highest value of the *VBc* parameter, equal to *VBc* = 0.381 mm (Figure 18).

**Figure 19:**
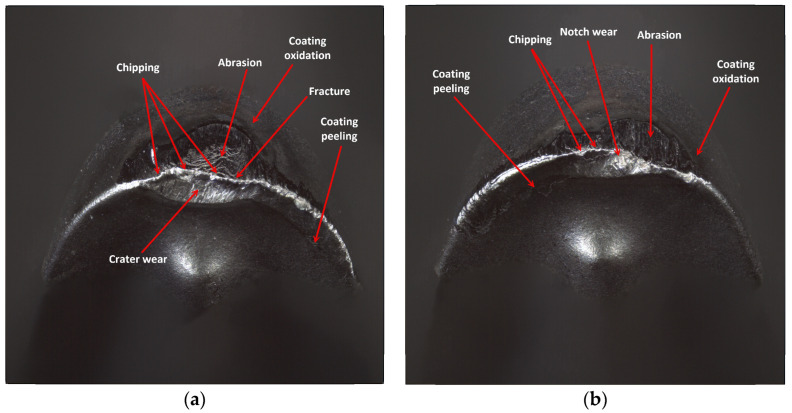
A view of the worn cutting edge of insert VCMT 160408–SM IC804: (**a**) an edge from a holder with a reach of 120 mm, (**b**) an edge from a holder with a reach of 700 mm.

The reason for the serious deterioration of the cutting edge is certainly the high value of the cutting speed [31], which in connection with the required cutting path, has contributed to the abrasive and strength wear of the edge. This statement is consistent with the findings of research conducted by Hao et al. [32]. In their study, the authors demonstrated that enhancement of the cutting speed to 45 m/min results in damage to the lubricating oxide film, leading to the formation of a large amount of worn tool material residue. Subsequently, oxidation reactions of tungsten (W) and cobalt (Co) occurred in the tool substrate material. The resulting oxides weakened the adhesion strength of cobalt (Co) and decreased the tool′s strength. It should be noted that the coating of that edge is one of less thickness, applied by the PVD method and, although the value of the cutting edge rounding radius was rather high, *r_n_* = 52.865 µm, the edge had not maintained the required strength at high temperatures. It should be taken into consideration that this edge has a larger flank angle, which also could influence its fatigue resistance. There was a meaningful decrement in the cutting edge, where chipping and fracture have also been recorded. The occurrence of coating peeling and oxidation has also been recorded, visible on the flank and the rake face (Figure 19). On the edges working in both holders notch and crater wear has arisen on the rake face due to chip flow. Considering the quality aspects, one can observe that such great deterioration of the edge has negatively influenced the obtained dimensional accuracy and surface quality.

**Figure 20:**
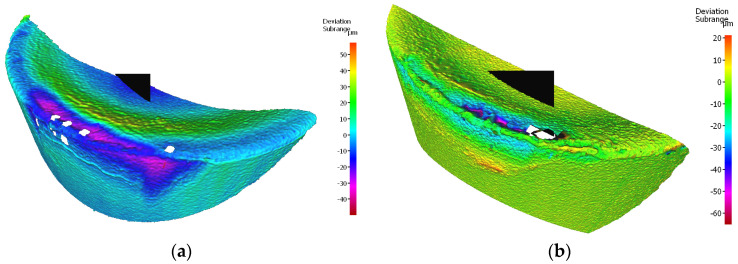
Topography of the worn cutting edge of the insert VBGT 160408–UM S205 after 19 min. of machining: (**a**) an edge from a holder with a reach of 120 mm, (**b**) an edge from a holder with a reach of 700 mm.

The edge having a lower value of the width of the flank wear, working in a holder of 120 mm reach, is the one of the cutting insert S205, for which *VBc* = 0.293 mm. For the edge of the same insert, but working in a holder with 700 mm reach, *VBc* = 0.200 mm (Figure 20).

**Figure 21:**
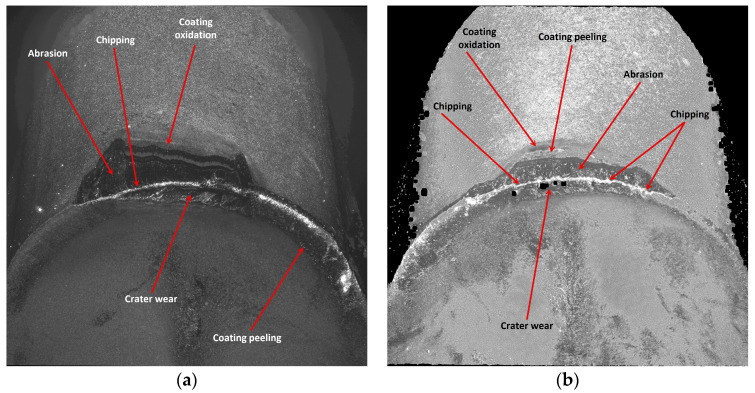
A view of the worn cutting edge of insert VBGT 160408–UM S205: (**a**) an edge from a holder with a reach of 120 mm, (**b**) an edge from a holder with a reach of 700 mm.

Analyzing the wear of the Sandvik insert, it is worth noticing that, in the edges working in both holders, mainly abrasive and strength character of the wear has occurred. Abrasive wear results from the presence of hard particles in the workpiece material, such as carbon, nitride and oxide compounds [8]. In the case of the edge working in the tool holder with 120 mm reach, a higher value of the abrasion wear on the flank face has occurred and crater wear on the rake face has started arising. Crater wear occurred as a result of chip flow, but may have been the beginning of diffusion wear. Unlike the PVD process, the CVD coating process may reduce the fracture toughness of the tool material under certain operating conditions due to the formation of cracks growing as a result of residual tensile stresses [8]. The phenomenon of crater wear is not so noticeable on the edge working in a holder with an overhang of 700 mm. On both edges, the phenomenon of coating oxidation and coating peeling can be observed. In both cases, chipping of the cutting edge occurred, a greater number of them were found on the edge working in the holder with 700 mm reach, resulting in its more irregular shape. This is undoubtedly due to the lower stability of the cutting process, due to the possible occurrence of small vibrations [8] (Figure 21).

According to Khochtali et al. [33], the increased number of chippings can be attributed to the combined mechanism of unstable build-up edge due to the unstable adhesion process and the accelerated adhesion process caused by the rapid removal of material deposited on the edge by a high-pressure water jet. Nevertheless, it should be noted that S205 is one of the relatively new coatings developed by Sandvik Company, Hong Kong, China. It is a strong CVD coating that is characterized by high abrasion resistance and hardiness to high temperatures in the cutting zone [34] at fairly high cutting speeds [35,36].

**Figure 22:**
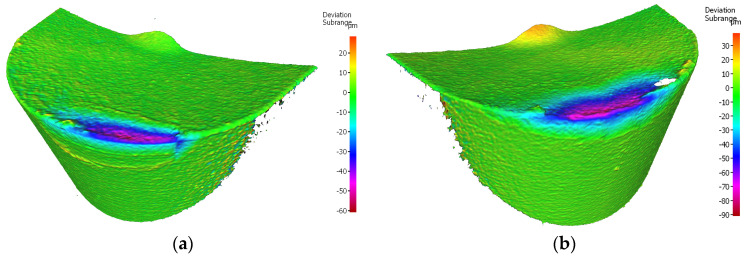
Topography of the worn cutting edge of the insert VCGT 160408–MM4 WSM01 after 19 min. of machining: (**a**) an edge from a holder with a reach of 120 mm, (**b**) an edge from a holder with a reach of 700 mm.

For the edges of insert WSM01, lower values of the width of the flank wear as compared to the edges of the previous inserts have been recorded. In the case of the cutting edge in the tool holder with 120 mm reach, *VBc* = 0.170 mm, for the edge working in a holder with 700 mm reach, *VBc* = 0.244 mm (Figure 22). The edges of that insert show relatively low values of wear in the case of both holders, considering the cutting path they have passed. The WSM01 coating is applied using the PVD method and considering information from the producer, it belongs to coatings with high abrasion resistance [37]. Under the influence of heat, aluminum is extracted from the TiAlN mesh to react with air, creating a passive and chemically stable Al_2_O_3_ oxide layer. The performance of this protective layer is further enhanced by high hot hardness and oxidation resistance (due to the creation of an intermediate layer consisting of titanium, aluminum, oxygen and nitrogen during processing). Another factor that can contribute to the high performance of the TiAlN coating is the presence of compressive residual stresses and their favorable ability to delay abrasive and notch wear [8]. In the case of edges that insert, built-up edges started appearing [38,39], regardless of the holder used. Inconel 718 material presents a high tendency to build up formation, which in the case of this insert was related to the evolution of adhesion wear intensity [40,41]. Adhesive wear is the result of high temperatures and pressures occurring during cutting, which causes welding between the clean chip surface and the rake face of the tool [8]. On both edges of that insert, the phenomenon of abrasion and strength wear have appeared. It should be stated that, on the edge working in the holder with 120 mm reach, less chipping has occurred, as compared to the edge working in the holder with 700 mm reach. Nevertheless, one should keep in mind that, on the edge working in a holder with shorter reach, crater wear and notch wear have appeared on the rake face. This kind of wear, located on the cutting edge nearer to the machined surface during the process of machining, combined with the largest width of flank wear, could have influenced the obtained higher surface roughness value.

**Figure 23:**
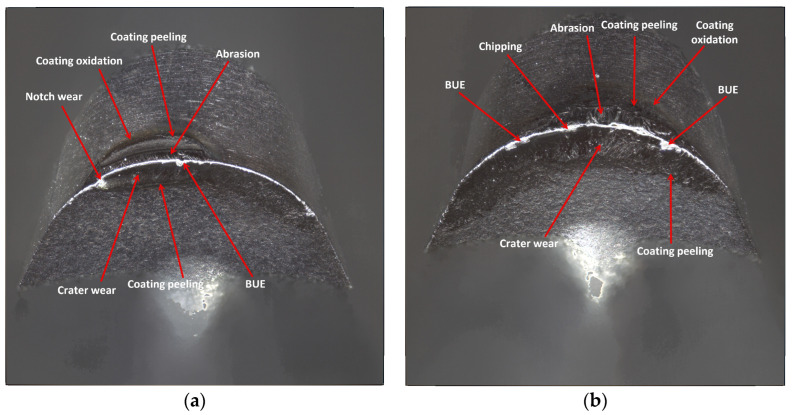
A view of the worn cutting edge of insert VCGT 160408–MM4 WSM01: (**a**) an edge from a holder with a reach of 120 mm, (**b**) an edge from a holder with a reach of 700 mm.

Oxidation and peeling of the coating also occurred on both edges of the WSM01 insert (Figure 23).

Obtaining a lower value of roughness of the surface for the edge working in the holder with 700 mm reach was most probably an effect of the more stable process of machining due to the sharp geometry of the insert as well as the deflection of the holder itself. The deflection has caused a reduction in the cross-section of the cut layer and consequently, a reduction in the load of the edge. The flank angle of the edge of that insert is 7°, and the cutting edge rounding radius *r_n_* = 12.44 µm which indicates a sharp edge.

**Figure 24:**
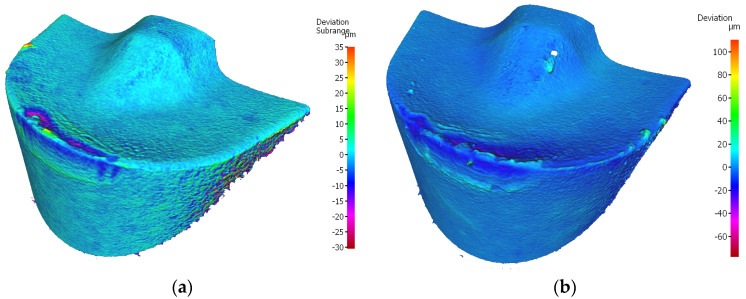
Topography of the worn cutting edge of the insert VBMT 160408–LS MP9005 after 19 min. of machining: (**a**) an edge from a holder with a reach of 120 mm, (**b**) an edge from a holder with a reach of 700 mm.

In the case of the edge of insert MP9005, for the holder with 120 mm reach, the height of the flank wear is *VBc* = 0.220 mm. For the edge of the insert working in the holder with 700 mm reach, *VBc* = 0.196 mm (Figure 24). It should be noted that, in the case of the edges of this insert, working in both holders, a built-up edge has been formed. The formation of built-up edge is on account of the fact that MP9005 is a new generation coating in the scope of higher abrasion resistance, but with a higher content of aluminum. The higher content of that element gives a better anti-friction effect in the initial phase of machining [42]. However, as tool wear evolves, it leads to the formation of aluminum oxides due to oxidation causing an increase in the coefficient of friction in tribological pairs [43]. This leads to increasing intensity of adhesion wear.

**Figure 25:**
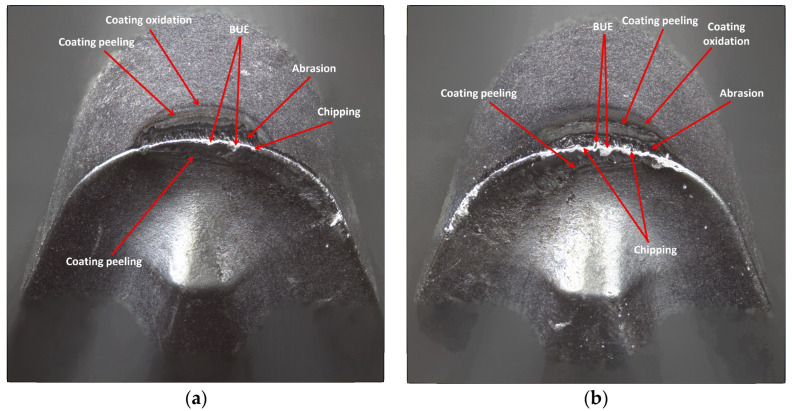
A view of the worn cutting edge of insert VBMT 160408–LS MP9005: (**a**) an edge from a holder with a reach of 120 mm, (**b**) an edge from a holder with a reach of 700 mm.

Nevertheless, the edge has high abrasion resistance which is evidenced by obtaining the lowest roughness value and the smallest deviation of the nominal dimension while working in stable conditions (holder with 120 mm reach). In the case of edges working in both holders forms of abrasion and strength wear have been recorded in the form of chipping on the cutting edge. However, it should be noted that on the cutting edge of the insert working in a tool holder with a reach of 700 mm, there was a much larger number of chipping which influenced the obtained surface roughness. This increased concentration of this kind of wear was caused by the lower stability of the process of machining as compared to machining with a shorter holder, as well as frequent sticking and falling off of adhesive materials leads to fluctuations in cutting forces and then causing chipping of the tool (Figure 25). This also leads to cracking between the tool coating and the tool substrate and then the PVD film is cut through the workpiece material and then peels off during the cutting process [32].

**Figure 26:**
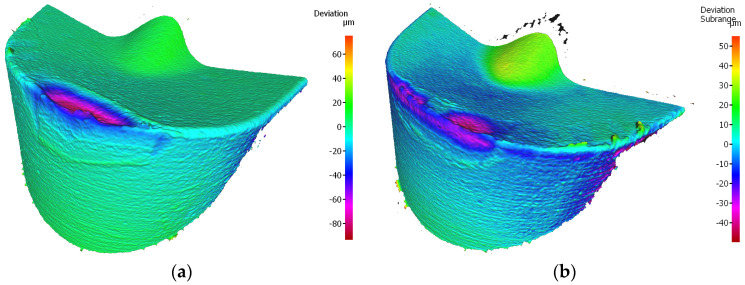
Topography of the worn cutting edge of the insert VBGT 160408LF KCS10B after 19 min. of machining: (**a**) an edge from a holder with a reach of 120 mm, (**b**) an edge from a holder with a reach of 700 mm.

Both cases were accompanied by the phenomenon of oxidation and peeling of the coating. Considering the wear of the cutting edges of insert KCS10B, for the edge working in a holder with 120 mm reach, the width of the flank wear is *VBc* = 0.230 mm. For the edge working in a holder with 700 mm reach, *VBc* = 0.107 mm (Figure 26).

**Figure 27:**
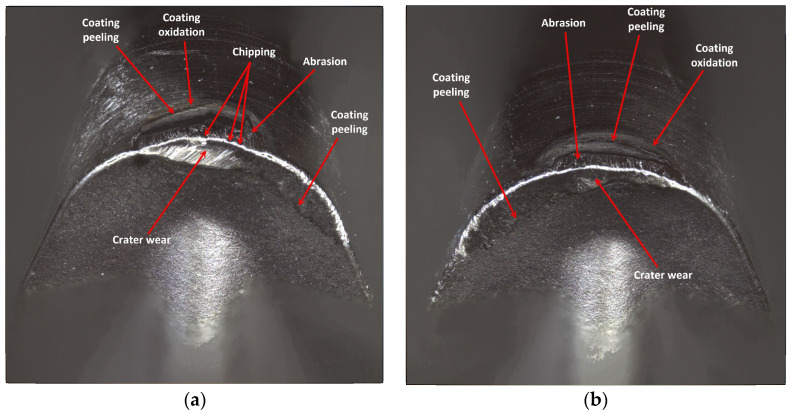
A view of the worn cutting edge of insert VBGT 160408LF KCS10B: (**a**) an edge from a holder with a reach of 120 mm, (**b**) an edge from a holder with a reach of 700 mm.

In the case of this insert, considering the value of the *VB_C_* parameter one can observe that its edges working on both holders have high strength and abrasion resistance. The KCS10B coating is a relatively new one applied using the PVD method and is characterized by resistance to high temperatures in the cutting zone, resulting from the high cutting speeds. On both edges, one can see abrasion and strength wear in the form of chipping. Crater wear on the rake face has also appeared, but a larger depth of it can be found on the edge working in a holder with 120 mm reach (Figure 27). Insert KCS10B also has sharp geometry because it is grind and its cutting edge rounding radius is *r_n_* = 29.944 µm.

It should be stated that, on the cutting edge of the insert which had worked in a holder with 700 mm reach, less chipping occurred as compared to the edge that had worked in a holder with 120 mm reach. This is most probably caused by the relatively sharp geometry of the insert obtained as a result of grinding, characterized by the low value of the radius of the cutting edge rounding *r_n_*, as well as by the deflection of the holder which has reduced the load of the edge and, at the same time damped the vibrations. The low concentration of the strength wear and the low value of the flank wear of that insert, working in a holder with 700 mm reach, has influenced the recorded lowest value of the surface roughness at the final stage of machining. This proves that the insert has well withstood such a long cutting path. Of course, in the case of both edges, the phenomenon of the coating peeling and oxidation took place. The KCS10B and MP9005 inserts were characterized by the lowest wear values evaluated by the parameter *VBc*, which was due to the PVD AlTiN coating, known for its high wear resistance. Confirmation of this can be found in the research results obtained by Zhang et al. [44]. They conducted machining studies on Inconel 718 alloy during turning at high cutting speeds (120, 160 and 200 m/min) using tools made of tungsten carbide with negative and positive edge geometries, coated with TiAlN, as well as tools made of tungsten carbide with a negative edge geometry, but coated with (Al, Ti) N, rich in aluminum. The research showed that inserts with a negative geometry coated with (Al, Ti) N have significantly longer tool life than inserts with TiAlN coating, both with positive and negative geometries, for all tested cutting speeds.

## 4. Conclusions

The conducted research aims to improve the process of finishing turning the internal surface with a long overhang tool for aircraft engine shaft class part.

Based on the investigation performed, the following conclusions have been formulated:

1. The reach of the tool holder influences the accuracy of the achieved dimensions and the quality of the surface under machining, as well as the edge wear. Depending on the tool holder used, the best results were recorded for MP9005, S205 and KCS10B edges, taking into account the achieved dimensional accuracy and the quality of the machined surface.
2. Considering the results of the obtained actual dimensions in relation to the nominal turned diameters, it should be stated that, for all the edges working in a tool holder, with 700 mm reach, larger deviations were obtained compared to the same edges working in a holder with 120 mm reach. At the terminal phase of machining, for the holder with 700 mm reach, the least value of deviation from the set dimension has been recorded for the surface machined with insert S205, the deviation was 0.21 mm. However, for a tool holder with an overhang of 120 mm, the smallest deviation value was noted for the area machined with the MP9005 insert, which was 0.01 mm. In the case of both holders, the highest values of the deviation from the assumed dimension have been recorded for the surfaces machined with the edges of insert IC804. Therefore, when a holder with a reach of 10 × D is used, it is necessary to consider the need for correction of the tool path. This is due to the deflection of the tool, the value of which increases with the progress of the edge wear.
3. The performed tests show that, for most edges, the longer reach of the tool had a negative influence on the obtained values of the surface topography and roughness. This is caused by the lower stability of the cutting process. In the case of the holder with 120 mm reach, the lowest values of roughness and topography of the surface at the terminal phase of machining were recorded for the surface cut with edge MP9005 and they were *Sa* = 0.952 µm, *Ra* = 0.924 µm. The highest values of the *Sa* and *Ra* parameters were registered for the area machined with insert IC804, where *Sa* = 2.606 µm and *Ra* = 2.302 µm. In the case of the tool holder with 700 mm overhang, the lowest values parameters of roughness and topography at the terminal phase of machining were recorded for an area machined with edge KCS10B and they were *Sa* = 1.253 µm, *Ra* = 0.804 µm. The highest values of the *Sa* and *Ra* parameters were noticed for an area machined with insert IC804, where *Sa* = 3.656 µm and *Ra* = 3.133 µm.
4. Taking into account the final stage of machining, regardless of the tool holder used, higher surface roughness and topography than predicted were recorded for all machined surfaces. This is undoubtedly caused by wear of the edges as a result of the relatively long operating time of the tools.
5. In the case of both holders, for the surfaces machined with the edges of insert IC804, the highest values of roughness and topography were recorded. The reason for obtaining such results was the largest wear of those edges evaluated by the *VBc* parameter and the highest value of the cutting edge rounding radius *r_n_*. Such a result confirms the consistency of the research with the results obtained by Fang [10] and Nieslony [27].
6. The conducted research clearly shows that some edges wear less when used in tools with reduced stiffness but are equipped with a vibration damper. This is undoubtedly an interesting area for further research. For the surfaces machined with edges WSM01 and KCS10B working in the holder with 700 mm reach, fewer values of roughness and topography have been recorded as compared to the edges working in a shorter holder. Such results were caused by the sharp geometry of the insert which, combined with the high strength of the cutting edge and deflection of the long holder resulted in less load on the edge. As a consequence, this has caused less flank wear of the edges under discussion and has changed the character of their wear. This has influenced the roughness and topography of the surface.
7. It should be noted that in the case of edges coated using the PVD method, the lowest wear was achieved for edges coated with the AlTiN coating, except for the IC804 edge. This coating is known for its longer durability compared to the traditional TiAlN coating. Regarding the IC804 edge, it should be emphasized that considering information from the manufacturer, this coating is intended for applications with lower and medium cutting speeds. This means that the cutting speed value used in the study may have been too high for this edge.
8. Analyzing the quality of the obtained surface, it is interesting that, in many cases regardless of the used tool holder, the values of surface roughness were highest in the third checkpoint and not at the final stage of machining. This point corresponds to the cutting path of 1049 m. This requires further investigation of the edge wear for defined checkpoints along the whole cutting path.

## Figures and Tables

**Table 1:** Register of tools utilized in the tests.

No.	Designation	Manufacturer	Type of Coating	Clearance Angle α [°]	Radius of the Cutting Edge Rounding, *r_n_* [µm]
**1**	VBGT 160408–UM S205	Sandvik Coromant (Sandviken, Sweden)	CVD (TiCN + Al_2_O_3_ + TiN)	5	35.127
**2**	VCGT 160408–MM4 WSM01	Walter (Tubingen, Germany)	PVD (TiAlN)	7	12.44
**3**	VBGT 160408LF KCS10B	Kennametal (Pittsburgh, PA, USA)	PVD (AlTiN)	5	29.944
**4**	VBMT 160408–LS MP9005	Mitsubishi Materials (Tokyo, Japan)	PVD (AlTiN)	5	27.045
**5**	VCMT 160408–SM IC804	Iscar Cutting tools (Tefen, Israel)	PVD (AlTiN)	7	52.865

**Table 2:** Average values of the outer diameter dimensions were obtained in the investigation for a holder with a reach of 120 mm.

No. of the Tool Pass/Cutting Path [m]	Nominal Diameter Dimension [mm]	Dimension Obtained after Machining with the S205 Insert [mm]	Dimension Obtained after Machining with the WSM01 Insert [mm]	Dimension Obtained after Machining with the KCS10B Insert [mm]	Dimension Obtained after Machining with the MP9005 Insert [mm]	Dimension Obtained after Machining with the IC804 Insert [mm]
2/235	131.2	131.218 ± 0.002	131.205 ± 0.002	131.2 ± 0.002	131.2 ± 0.002	131.2 ± 0.002
5/586	130	130.027 ± 0.003	130.012 ± 0.002	130.004 ± 0.002	130.001 ± 0.002	130.005 ± 0.002
9/1049	128.4	128.444 ± 0.003	128.42 ± 0.003	128.413 ± 0.002	128.404 ± 0.002	128.417 ± 0.002
14/1620	126.4	126.462 ± 0.004	126.43 ± 0.003	126.422 ± 0.003	126.41 ± 0.003	126.465 ± 0.004

**Table 3:** Average values of the internal diameter dimensions obtained in the investigation for a holder with a reach of 700 mm.

No. of the Tool Pass/Cutting Path [m]	Nominal Diameter Dimension [mm]	Dimension Obtained after Machining with the S205 Insert [mm]	Dimension Obtained after Machining with the WSM01 Insert [mm]	Dimension Obtained after Machining with the KCS10B Insert [mm]	Dimension Obtained after Machining with the MP9005 Insert [mm]	Dimension Obtained after Machining with the IC804 Insert [mm]
2/235	78.2	78.15 ± 0.005	78.17 ± 0.005	78.17 ± 0.005	78.19 ± 0.005	78.0 ± 0.005
5/586	79.4	79.30 ± 0.005	79.265 ± 0.005	79.32 ± 0.005	79.32 ± 0.005	79.15 ± 0.01
9/1049	81	80.86 ± 0.01	80.79 ± 0.01	80.84 ± 0.01	80.85 ± 0.01	80.60 ± 0.01
14/1620	83	82.79 ± 0.01	82.65 ± 0.01	82.76 ± 0.01	82.75 ± 0.01	82.28 ± 0.015

## Data Availability

Dataset available on request from the authors.
